# Slow‐decaying presynaptic calcium dynamics gate long‐lasting asynchronous release at the hippocampal mossy fiber to CA3 pyramidal cell synapse

**DOI:** 10.1002/syn.22178

**Published:** 2020-07-16

**Authors:** Simon Chamberland, Yulia Timofeeva, Alesya Evstratova, Christopher A. Norman, Kirill Volynski, Katalin Tóth

**Affiliations:** ^1^ CERVO Brain Research Center Department of Psychiatry and Neuroscience Université Laval Quebec QC Canada; ^2^ Department of Computer Science University of Warwick Coventry UK; ^3^ Centre for Complexity Science University of Warwick Coventry UK; ^4^ University College London Institute of Neurology University College London London UK; ^5^ Mathematics for Real‐World Systems Centre for Doctoral Training University of Warwick Coventry UK; ^6^ Department of Cellular and Molecular Medicine Faculty of Medicine University of Ottawa Ottawa ON Canada

**Keywords:** asynchronous release, calcium, hippocampus, mossy fibers, presynaptic

## Abstract

Action potentials trigger two modes of neurotransmitter release, with a fast synchronous component and a temporally delayed asynchronous release. Asynchronous release contributes to information transfer at synapses, including at the hippocampal mossy fiber (MF) to CA3 pyramidal cell synapse where it controls the timing of postsynaptic CA3 pyramidal neuron firing. Here, we identified and characterized the main determinants of asynchronous release at the MF–CA3 synapse. We found that asynchronous release at MF–CA3 synapses can last on the order of seconds following repetitive MF stimulation. Elevating the stimulation frequency or the external Ca^2+^ concentration increased the rate of asynchronous release, thus, arguing that presynaptic Ca^2+^ dynamics is the major determinant of asynchronous release rate. Direct MF bouton Ca^2+^ imaging revealed slow Ca^2+^ decay kinetics of action potential (AP) burst‐evoked Ca^2+^ transients. Finally, we observed that asynchronous release was preferentially mediated by Ca^2+^ influx through P/Q‐type voltage‐gated Ca^2+^ channels, while the contribution of N‐type VGCCs was limited. Overall, our results uncover the determinants of long‐lasting asynchronous release from MF terminals and suggest that asynchronous release could influence CA3 pyramidal cell firing up to seconds following termination of granule cell bursting.

## Graphical abstract

Asynchronous release at MF‐CA3 synapses can last on the order of seconds and presynaptic Ca^2+^ dynamics are the major determinants of this type of release rate. Asynchronous release was preferentially mediated by P/Q‐type voltage‐gated Ca^2+^ channels, while the contribution of N‐type VGCCs was limited.

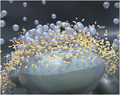

## 1 INTRODUCTION

Neurotransmitter release from presynaptic terminals occurs in different modes following action potential firing. Action potentials propagating along the axon reach the presynaptic terminal and generate Ca^2+^ influx, which causes vesicle fusion and neurotransmitter release. Synchronous and asynchronous release both depend on a common presynaptic Ca^2+^ influx, but with different dependency on the Ca^2+^ concentration (Atluri & Regehr, 1998). Synchronous and asynchronous release co‐occur at multiple synapses, albeit on different timescales (Atluri & Regehr, 1998; Iremonger & Bains, 2007). Synchronous release is precisely timed to the action potential‐induced Ca^2+^ influx, while asynchronous release is temporally delayed, and can last for hundreds of milliseconds following stimulus termination (Barrett & Stevens, 1972; Daw, Tricoire, Erdelyi, Szabo, & McBain, 2009; Hefft & Jonas, 2005). The high speed and reliability of synchronous release is thought to be crucial for the ability of neuronal networks to rapidly process information, while the role of asynchronous release remains generally mysterious. At a number of synapses, asynchronous release contributes to the neuronal dialog by controlling the precise timing of postsynaptic firing (Evstratova, Chamberland, Faundez, & Toth, 2014; Iremonger & Bains, 2007; Luo & Sudhof, 2017). Thus, understanding the dynamics of both synchronous and asynchronous release are important to obtain a comprehensive picture of information transfer at different types of brain synapses (Rozov, Bolshakov, & Valiullina‐Rakhmatullina, 2019). However, the key determinants of asynchronous release remain mostly ill‐defined.

Granule cells receive extra‐hippocampal excitatory afferents from the entorhinal cortex. In turn, granule cells project their mossy fiber (MF) axons to hippocampal CA3 pyramidal cells. Granule cells can fire action potentials in bursts (Jung & McNaughton, 1993; Pernia‐Andrade & Jonas, 2014), which are sufficient to trigger CA3 pyramidal cell firing (Henze, Wittner, & Buzsaki, 2002). Synapses formed between the MF axons of hippocampal granule cells and CA3 pyramidal cells are unique relays of information (Amaral & Dent, 1981). The presynaptic terminals of these structures incorporate multiple release sites (18–45) (Rollenhagen et al., 2007), which are apposed to dendritic specializations called thorny excrescences (Chicurel & Harris, 1992). Moreover, MF terminals possess a large number of synaptic vesicles (Hallermann, Pawlu, Jonas, & Heckmann, 2003). Functionally, this peculiar arrangement allows for the large dynamic range of neurotransmitter release observed at this synapse. Synchronous and asynchronous release are known to co‐occur from a large number of presynaptic terminals, including the MF synapse (Evstratova et al., 2014; Evstratova & Toth, 2014). However, it is unknown whether these two components of release can be selectively recruited by different patterns of activity.

The MF–CA3 synapse has long been recognized as a “detonator” synapse, owing to the observation that a single action potential (AP) from a granule cell can trigger postsynaptic CA3 spiking (Urban, Henze, & Barrionuevo, 2001), under condition of sufficient glutamate release probability (Vyleta, Borges‐Merjane, & Jonas, 2016). Synchronous glutamate release is vastly amplified during repetitive trains of APs through a combination of multiple mechanisms (Chamberland, Evstratova, & Toth, 2014, 2017; Evstratova & Toth, 2014; Vyleta & Jonas, 2014). Interestingly, the combination of these short‐term facilitation mechanisms results in a peculiar information transfer strategy. Information transfer at the MF–CA3 synapse relies on a code that is frequency‐independent, but rather counts the number of action potential in the regime of 10–100 Hz (Chamberland, Timofeeva, Evstratova, Volynski, & Toth, 2018). How the frequency of presynaptic APs contribute to neuronal communication, as at most synapses, remains unknown. Furthermore, how specific patterns of presynaptic action potential firing can enhance or optimize asynchronous release remains unknown.

In giant MF terminals, action potential‐evoked Ca^2+^ influx is mediated by P/Q‐, N‐, and R‐type VGCCs (Chamberland et al., 2017; Li, Bischofberger, & Jonas, 2007). P/Q‐ and N‐type VGCCs both contribute to synchronous glutamate release, and the simultaneous blockade of these two type of channels is sufficient to abolish synchronous release, even during repetitive stimulation (Castillo, Weisskopf, & Nicoll, 1994; Chamberland et al., 2017). P/Q‐type VGCCs mediate the largest fraction of Ca^2+^ influx in MF boutons (Chamberland et al., 2017; Li et al., 2007), consistent with the observations that they are the main contributor to synchronous glutamate release (Castillo et al., 1994; Chamberland et al., 2017; Pelkey, Topolnik, Lacaille, & McBain, 2006). Furthermore, P/Q‐ and N‐type VGCCs generate distinct spatial patterns of Ca^2+^ elevations and VGCCs are in a loosely coupled configuration to vesicular Ca^2+^ sensors (Chamberland et al., 2017). Interestingly, this coupling configuration has been previously associated with enhanced levels of asynchronous release (Hefft & Jonas, 2005) and contributes to short‐term facilitation in MF synapses (Vyleta & Jonas, 2014). Last, Ca^2+^ elevations are slowly decaying in MF terminals (Chamberland et al., 2018; Scott & Rusakov, 2006), in part owing to elevated presynaptic Ca^2+^ buffering (Vyleta & Jonas, 2014). How slowly decaying presynaptic Ca^2+^ dynamics (shaped by Ca^2+^ extrusion and buffering) contribute to asynchronous glutamate release from MF remains unexplored.

Here, we report that asynchronous release is long lasting at the MF–CA3 synapse. The frequency of presynaptic APs is the major factor that determines presynaptic Ca^2+^ dynamics to tune the amount of asynchronous release at MF terminals. Furthermore, we show that Ca^2+^ influx through P/Q‐type but not N‐type VGCCs significantly contributes to asynchronous release at this synapse.

## 2 RESULTS

### 2.1 Asynchronous release is long‐lasting at MF–CA3 synapses

Asynchronous release occurs following presynaptic firing and can last on the order of hundreds of milliseconds following stimulus termination. However, the properties of asynchronous release at MF–CA3 pyramidal cells remain generally undefined. We first aimed to investigate the dynamics of asynchronous release at MF–CA3 synapses.

**FIGURE 1:**
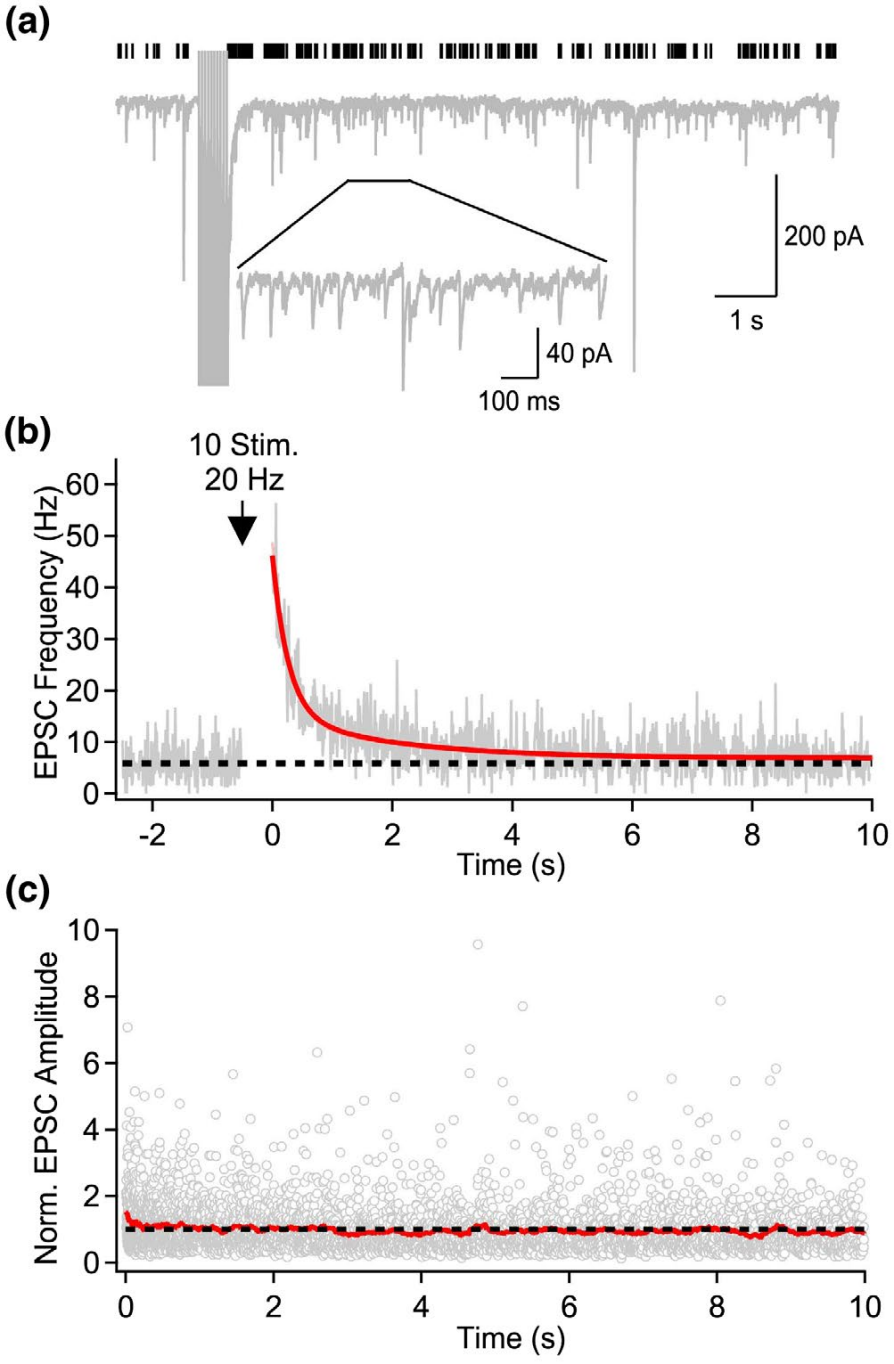
Long‐lasting asynchronous release at MF–CA3 synapses. (a) Example voltage‐clamp recording from a CA3 pyramidal cell performed in 6 mM external Ca^2+^, to maximize asynchronous release. MF were stimulated 10 times at 20 Hz. Black ticks above the trace indicate the presence of automatically detected EPSC. (b) Time course of spontaneous EPSC frequency (10 ms bins) before and after the stimulation train indicated by the black arrow (20 Hz, 10 stimuli). The rate of asynchronous release was quantified by measuring the number of asynchronous EPSCs in a 10 s window starting 10 ms after the peak of the last stimulus in the train. The red line corresponds to a bi‐exponential fit of the average data (*n* = 7 neurons). (c) Normalized amplitude of detected asynchronous EPSCs as a function of time. EPSCs amplitude were normalized in individual neurons before averaging all cells together (*n* = 7 neurons). The red line is a moving average

To capture the full temporal dynamics of asynchronous release, MF axons were electrically stimulated under high release conditions imposed by artificially elevating the extracellular Ca^2+^ concentration to 6 mM while voltage‐clamp recordings were performed from CA3 pyramidal cells. About 10 stimuli were delivered at 20 Hz, following which asynchronous EPSCs were automatically detected 10 ms following the last stimulus in the train (Figure 1a‐b). Quantification of the EPSC frequency in 10 ms bins revealed a ~ fivefold increase in EPSC frequency following the stimulus train (*n* = 7 neurons). This increase in EPSC frequency lasted on the order of seconds (Figure 1b). Consistent with the quantal nature of asynchronous EPSCs, the EPSC amplitude remained constant over 10 s following stimuli train (Figure 1c). Therefore, these results indicate that asynchronous release from MF terminals is long‐lasting and maintained for seconds following stimuli termination under high release probability.

### 2.2 High‐frequency activity favors asynchronous release

Dentate granule cells fire action potentials with varying frequencies (Pernia‐Andrade & Jonas, 2014). Synchronous and asynchronous release co‐occur in presynaptic terminals, including from MFBs (Evstratova et al., 2014). However, it remains unknown whether different patterns of activity selectively recruit distinct modes of release and how the stimulation frequency modulates asynchronous release from MFBs.

**FIGURE 2:**
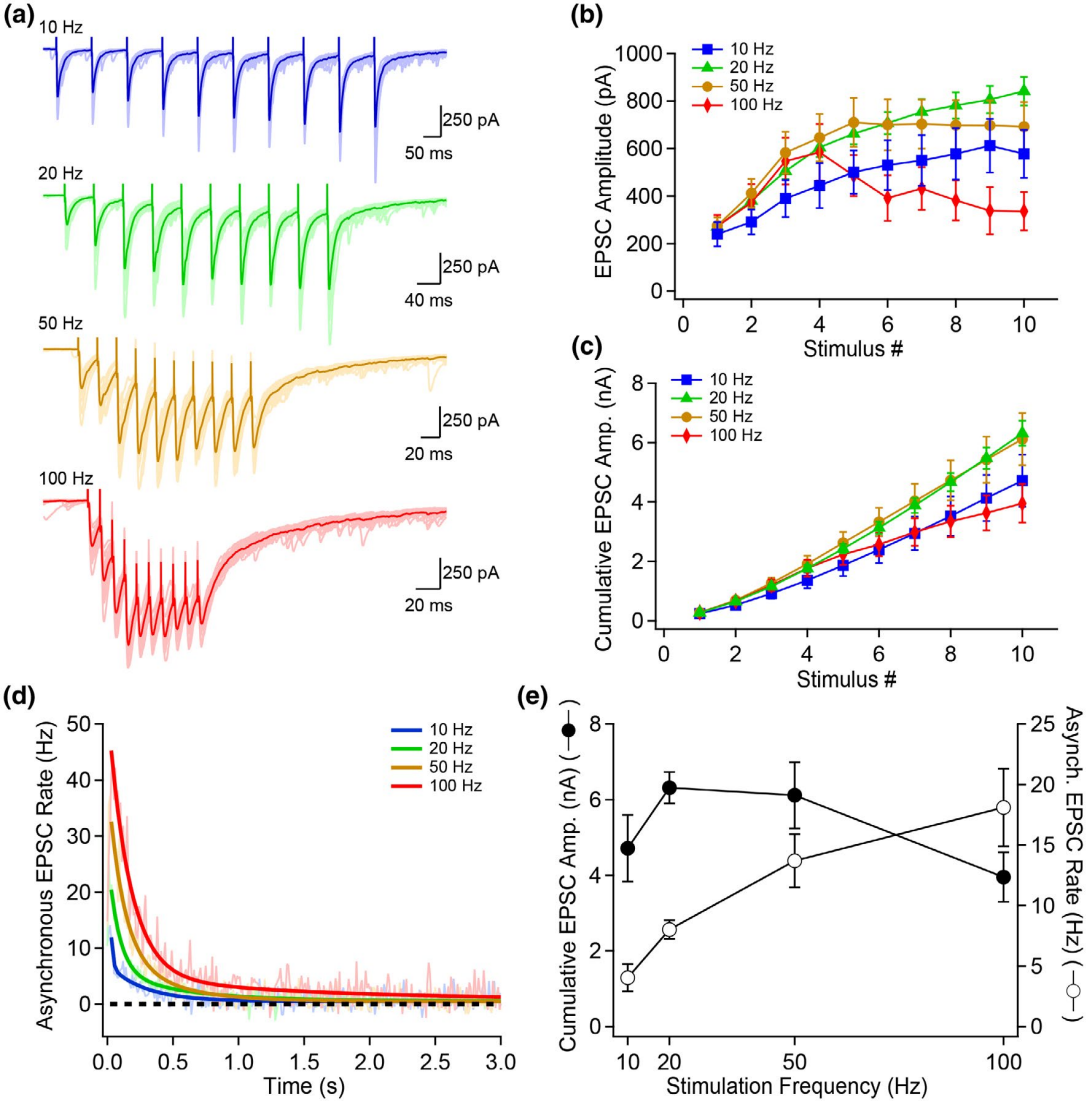
High frequency firing selectively recruits asynchronous over synchronous release. (a) Trains of MF–EPSCs evoked by 10 stimuli at 10, 20, 50 or 100 Hz in ACSF containing 2.5 mM Ca^2+^. Data shown are 20 consecutive trials with overlaid average. Asynchronous EPSCs were detected as described in Figure 1. (b) Synchronous EPSC amplitude as a function of stimulus number for the four frequencies shown in (a). (c) Cumulative synchronous EPSC amplitude as a function of stimulus number for 10, 20, 50, and 100 Hz stimulation. (d) Quantification of asynchronous release as a function of time (10 ms bins) for 10, 20, 50, and 100 Hz stimulation. The full lines correspond to bi‐exponential fits of the average data. (e) Cumulative synchronous EPSC amplitude and asynchronous EPSC rate as a function of stimulation frequency, showing that synchronous and asynchronous release are differentially recruited by different stimulation frequencies, with asynchronous release being especially sensitive to stimulation frequency. Asynchronous release was measured over a 500 ms window starting 10 ms after the last stimulus. Number of replicates: *n* = 10 for 10 Hz; *n* = 48 for 20 Hz; *n* = 13 for 50 Hz; *n* = 8 for 100 Hz

We recorded trains of MF–EPSCs evoked by 10 stimuli at 10, 20, 50, or 100 Hz (Figure 2a‐b) in artificial cerebrospinal fluid (ACSF) containing 2.5 mM Ca^2+^. Quantification of synchronous release revealed that the progression of EPSC amplitude as a function of stimulus number was strongly dependent on the stimulation frequency (Figure 2b). Short‐term facilitation was readily observed at all stimulation frequencies and was generally more pronounced for higher frequencies (Figure 2b). While the 50 Hz trains resulted in a plateau portion for the last six stimuli, the 100 Hz stimulation generated EPSCs that declined in amplitude for the last six stimuli. Consistent with this result, the cumulative synchronous EPSC amplitude associated with 10 Hz stimulation fell below the cumulative EPSC amplitude recorded with 20 and 50 Hz stimulation (Figure 2c). On the contrary, the 100 Hz stimulation resulted in a ~40% decrease in total release by the 10th stimulus when compared to the 20 Hz train. These results indicate that synchronous release is initially favored by higher stimulation frequencies, but vesicle release fails to increase at 50 Hz and declines after the 4th stimulus at 100 Hz.

Next, we took advantage of this finding to examine if synchronous and asynchronous release similarly depend on the stimulation pattern. The rate of asynchronous release was dependent on the stimulation frequency (Figure 2d) and increased as a function of the stimulation frequency (Figure 2e) when the analysis was focused on a 500 ms window starting 10 ms following the last stimulation. Compared to the cumulative synchronous release, which displayed a biphasic shape as a function of the stimulation frequency, asynchronous release was consistently increased as a function of the stimulation frequency for sustained activity (Figure 2e). Overall, our results indicate that synchronous and asynchronous release operate independently as a function of stimulation intensity, with higher frequencies favoring asynchronous release.

### 2.3 Asynchronous release depends on external Ca^2+^ concentration

The above results revealed the frequency‐dependence of asynchronous release. We hypothesized that this phenomenon could be due, at least in part, to higher amplitudes presynaptic Ca^2+^ elevations. As in other synapses, this would suggest that asynchronous release is dependent on the integrated Ca^2+^ concentration over time.

**FIGURE 3:**
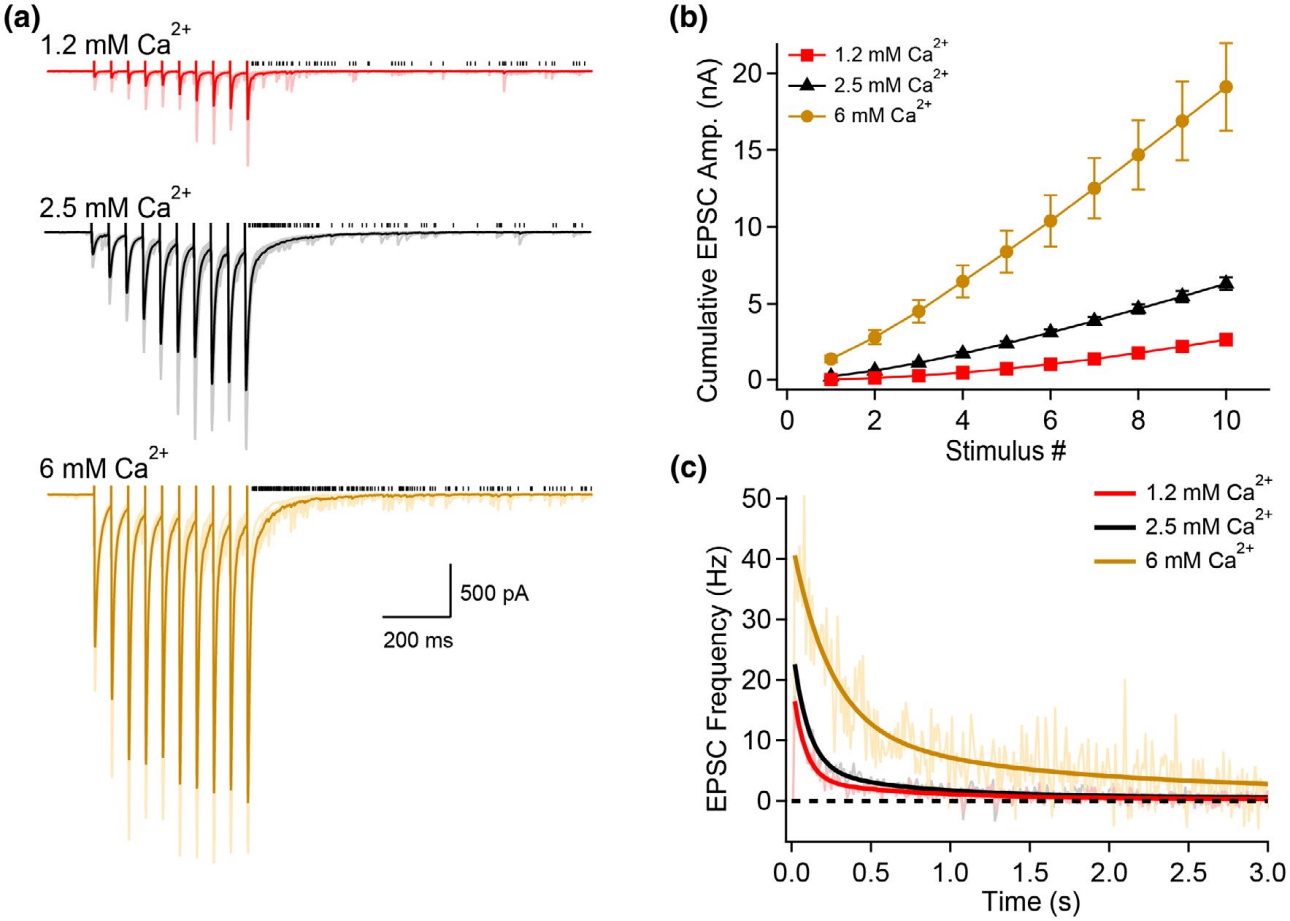
Asynchronous release strongly depends on external Ca^2+^. (a) Example voltage‐clamp recordings of synchronous and asynchronous EPSCs evoked in 1.2, 2.5, and 6 mM external Ca^2+^. Data shown correspond to 20 consecutive trials, with overlaid average. Black ticks show the detected asynchronous EPSCs. (b) Cumulative synchronous EPSCs as a function of stimulus number for recordings performed in 1.2, 2.5, and 6 mM external Ca^2+^. (c) Asynchronous release as a function of time following the termination of the stimuli train. Full lines correspond to bi‐exponential fits of the average data. Number of replicates: *n* = 35 for 1.2 mM Ca^2+^; *n* = 48 for 2.5 mM Ca^2+^; *n* = 7 for 6 mM Ca^2+^

To test this idea, the stimulation frequency was kept constant (20 Hz) while the external Ca^2+^ concentration was varied (Figure 3a). We observed that both synchronous and asynchronous release were elevated by increasing the external Ca^2+^ concentration (Figure 3b‐c). Therefore, the frequency‐dependence of asynchronous release is likely to be explained by a higher presynaptic Ca^2+^ concentration.

### 2.4 Bursts of action potentials evoke invariant and long‐lasting Ca^2+^ transients in MF boutons

Asynchronous release strongly depends on presynaptic Ca^2+^ and the stimulation frequency. To understand the presynaptic Ca^2+^ dynamics gating asynchronous release, we next quantified the temporal dynamics of single action potential‐ and burst‐evoked Ca^2+^ elevations in MF boutons.

**FIGURE 4:**
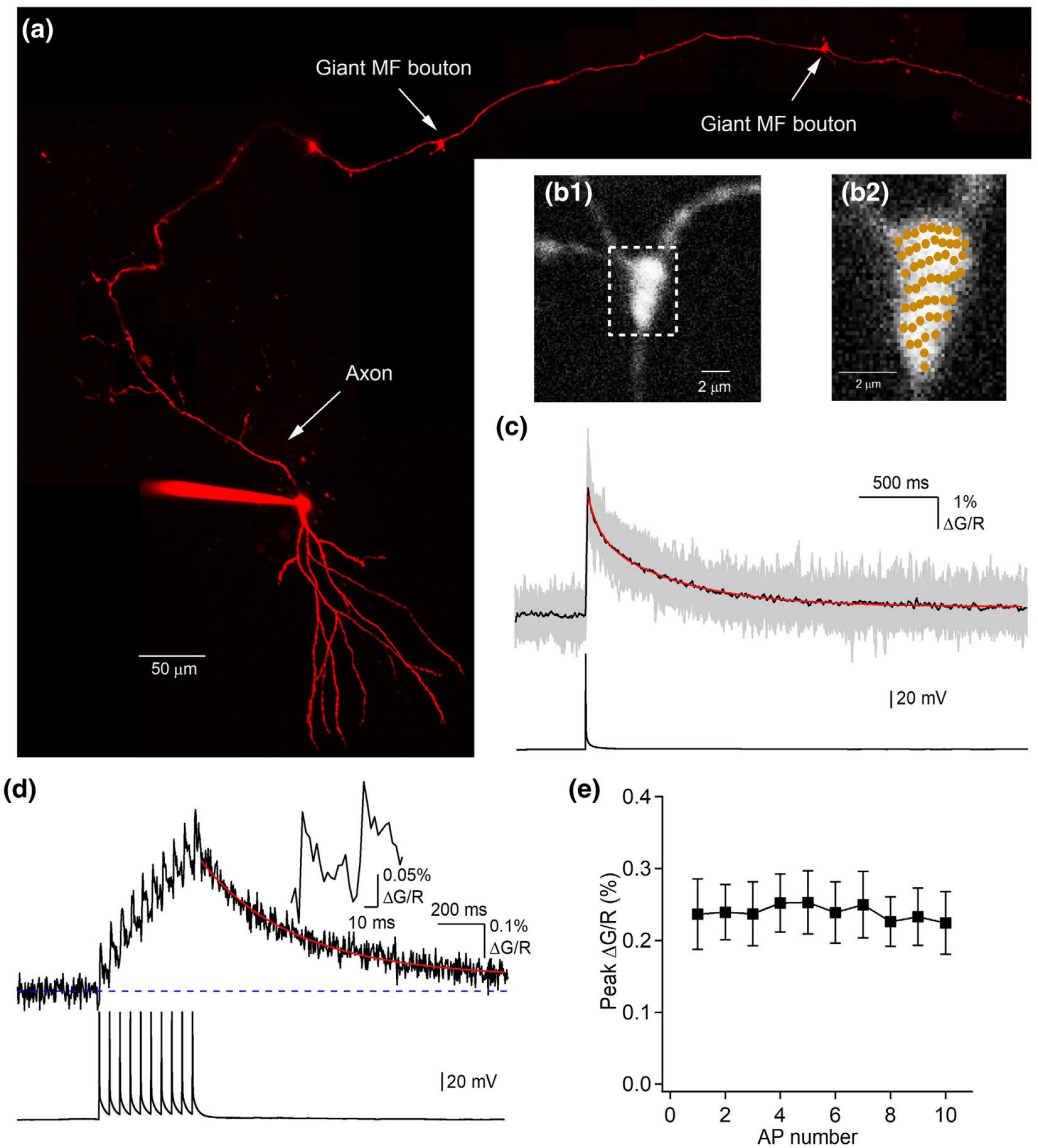
Action potentials evoke slow‐decaying Ca^2+^ transients in MF boutons. (a) Granule cell filled with the morphological indicator Alexa‐594 and imaged with two‐photon microscopy. This image is a collage from multiple Z‐stacks maximal projections. (b) Zoomed‐in image showing a giant MF bouton filled with Alexa‐594 (b1) and the recording sites (b2) for the data shown below. (c) Representative Ca^2+^ transients recorded using Fluo‐5F from multiple points and averaged over 20 trials (individual grey traces). Black trace is the spatial average. The red line is a bi‐exponential fit to the decay portion of the average Ca^2+^ transient. Current‐evoked action potential is shown below. (d) Ca^2+^ transient reported by Fluo‐4FF and evoked by a burst of 10 APs generated at 20 Hz (shown below). The red trace is the fit from the single‐compartment model. (e), Peak individual Ca^2+^ transients as a function of AP number. The peak amplitude does not change as a function of AP number

Individual MF boutons from granule cells axons were identified following whole‐cell loading of the morphological indicator Alexa‐594 for at least 1 hr (Figure 4a). Presynaptic Ca^2+^ dynamics recordings were obtained using random access two‐photon imaging, allowing recordings with both high spatial and temporal resolutions (Figure 4b1,2). Single APs were evoked by current injection in the granule cells through the patch pipette and associated Ca^2+^ transients were measured using the Ca^2+^ indicator Fluo‐5F (385 µM) (Figure 4c). Under this recording condition, Ca^2+^ transients had an average amplitude of 5.24 ± 0.53% ΔG/R (*n* = 14) with a fast rise time (11.93 ± 0.27 ms, *n* = 14) and slow decay kinetics (decay τ: 491.86 ± 37.7 ms, *n* = 14) a finding consistent with presynaptic terminals of large volume (Zhang & Linden, 2012). Therefore, single action potentials evoke long‐lasting Ca^2+^ transients in MF boutons, the speed of which is likely dictated by a slow extrusion process (Scott & Rusakov, 2006). We next measured how individual Ca^2+^ transients vary and summate during repetitive activity to generate asynchronous release, using experimentally constrained modeling. Our previous results showed that amplitudes of individual Ca^2+^ elevations recorded with Fluo‐4FF in MF boutons are invariant during trains (Chamberland et al., 2018), in recording conditions where the ACSF contained 1.2 mM Ca^2+^. We performed presynaptic Ca^2+^ imaging during trains of APs using the low affinity Ca^2+^ indicator Fluo‐4FF in conditions where the ACSF contained 2.5 mM Ca^2+^ (Figure 4d). Following 10 APs evoked at 20 Hz, we observed that AP‐evoked Ca^2+^ transient amplitudes were invariant during trains (*n* = 11 boutons), in line with our previous results (Chamberland et al., 2018) (Figure 4e).

We next fitted the decay kinetics of burst‐evoked Ca^2+^ transients using the single‐compartment model (methods) in order to be able to model AP‐evoked presynaptic Ca^2+^ dynamics at different stimulation patterns under dye‐free conditions. We observed that the decay time of burst‐evoked presynaptic Ca^2+^ transients was slow, with Ca^2+^ removal rate constant in the range of 0.2–0.4 ms^‐1^ (0.26 ms^−1^ for the example shown in Figure 4d). This result argues that the slowly decaying Ca^2+^ elevations in giant MF boutons could indeed represent a mechanism for the generation and maintenance of long‐lasting asynchronous release.

### 2.5 Experimentally constrained modeling of asynchronous release shows close dependency on presynaptic Ca^2+^

**FIGURE 5:**
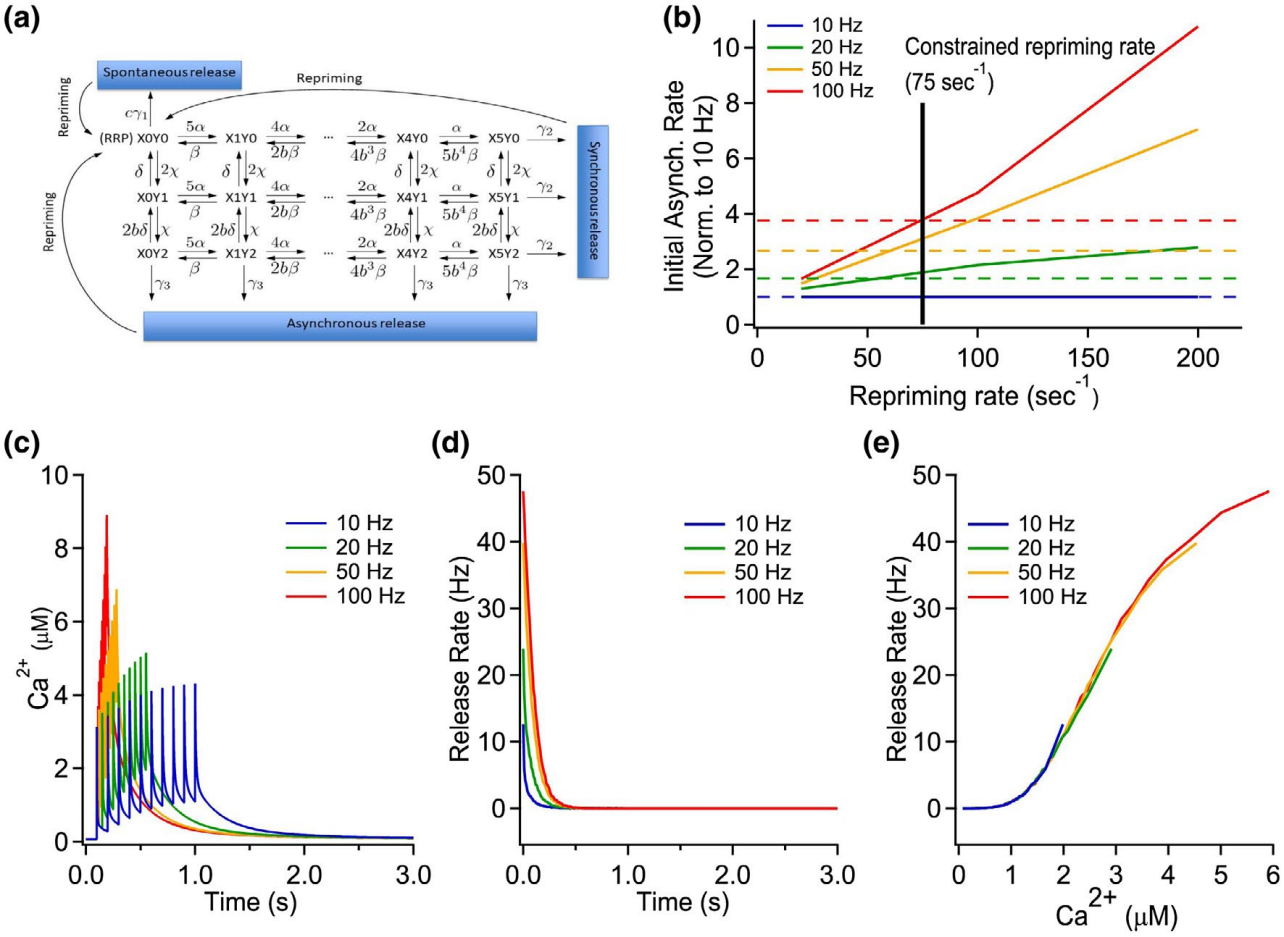
A dual‐sensor model of Ca^2+^‐driven vesicle release replicates the frequency‐dependence of asynchronous release. (a) Schematics of the dual‐sensor model of Ca^2+^‐driven vesicle release with additional repriming dynamics (Sun et al., 2007). (b) Constraining of the repriming rate in the modified dual‐sensor model. The model was initially computed with the instantaneous repriming rate as well as with the rates of 20 and 100 s^−1^. Release rate values at the start of asynchronous release normalized to the 10 Hz stimulation scenario were then compared with the corresponding experimental release rate values. This analysis suggested of using the release rate of 75 s^−1^ in the results simulations. (c) Ca^2+^ traces computed in the nonstationary single‐compartment model for each stimulation paradigm and used in the dual‐sensor release model. (d) Computed release rates of a readily releasable pool (RRP) of 100 vesicles for each stimulation paradigm. (e) Vesicular release rate (of RRP = 100 vesicles) as a function of [Ca^2+^]

Next, we performed computational modeling of Ca^2+^ dynamics and neurotransmitter release. Here, we utilized a nonstationary single compartment model (described in Material and Methods) used by us in (Chamberland et al., 2018) for simulating the presynaptic Ca^2+^ dynamics. Consistent with experimental data analysis, asynchronous release was considered to occur 10 ms after the last AP, by which time the spatial gradient of Ca^2+^ dynamics in the terminal disappears, justifying the application of a single compartment model in this study (instead of spatially extended three‐dimensional models). We assumed the presence of three major endogenous Ca^2+^ buffers (calbindin‐D_28K_, calmodulin, and ATP) and computed Ca^2+^ traces for the four different stimulation scenarios (Figure 5c). The peak Ca^2+^ transients increased as a function of the stimulation frequency. The obtained [Ca^2+^] transients were then used in the so‐called dual‐Ca^2+^‐sensor model for neurotransmitter release (Sun et al., 2007) that was originally developed to describe the contributions of synchronous and asynchronous release events at the Calyx of Held. As shown in Figure 5a, we modified this model to take into account vesicle replenishment by introducing additional repriming steps. The repriming rate was constrained to the value *K_rep_* = 75 s^−1^ by performing simulations for a number of different rate constants and comparing the initial asynchronous release rates with experimental data (Figure 5b). The dual‐sensor release model was implemented using a stochastic numerical algorithm (Anderson, 2007; Gillepsie, 1977), and the rate of asynchronous release was plotted in Figure 5d indicating a similarity with the experimental data. In Figure 5e, we also compared the dependency of asynchronous release rates on [Ca^2+^] triggered by AP bursts of different frequency. Overall, the modeling results replicate the frequency‐dependence of asynchronous release after the end of the stimuli, and confirm the dependence of asynchronous release on presynaptic Ca^2+^ concentration.

### 2.6 Distinct contribution of P/Q‐ and N‐type VGCCs to AP‐evoked presynaptic Ca^2+^ transients and release

VGCCs are the main mediators of AP‐evoked presynaptic Ca^2+^ influx. Considering the strong dependence of asynchronous release on presynaptic Ca^2+^, we next aimed to understand how two types of VGCCs contribute to presynaptic Ca^2+^ influx and asynchronous release.

**FIGURE 6:**
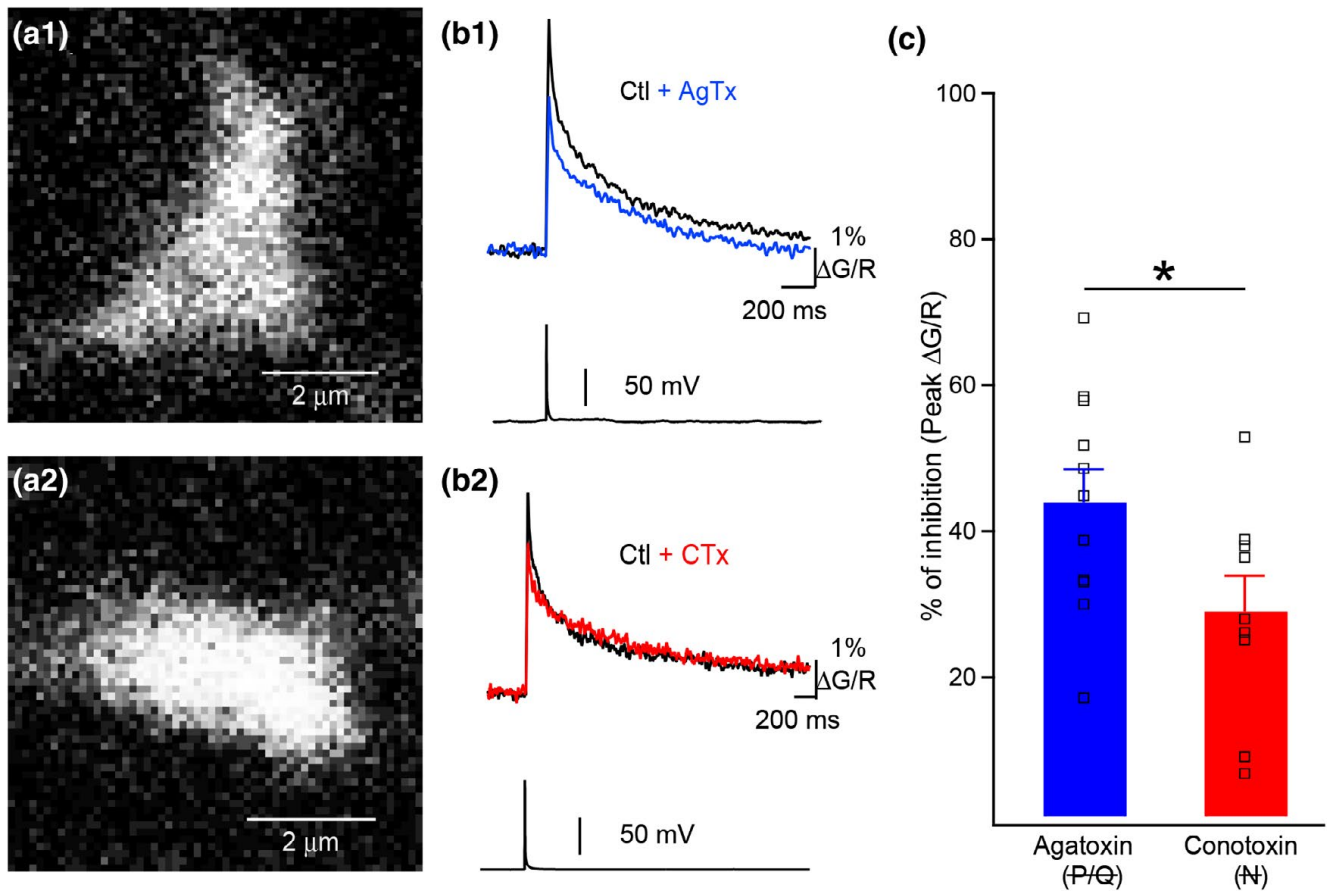
Larger contribution of P/Q‐ than N‐type VGCCs to Ca2 + influx in mossy fiber boutons. (a1, a2) Giant MF boutons imaged with Alexa‐594 from which the data in B was obtained. (b) Representative AP‐evoked Ca^2+^ transients recorded with Fluo‐5F in control condition (black) and following bath application of AgTx (b1, blue trace) and CTx (b2, red trace). (c) Summary graph showing the percentage of peak Ca^2+^ transient inhibition caused by AgTx (blue) and CTx (red). These recordings were performed in 1.2 and 2.5 mM external Ca^2+^. Overlaid squares correspond to individual boutons. *p < .05, unpaired Student's *t* test

**FIGURE 7:**
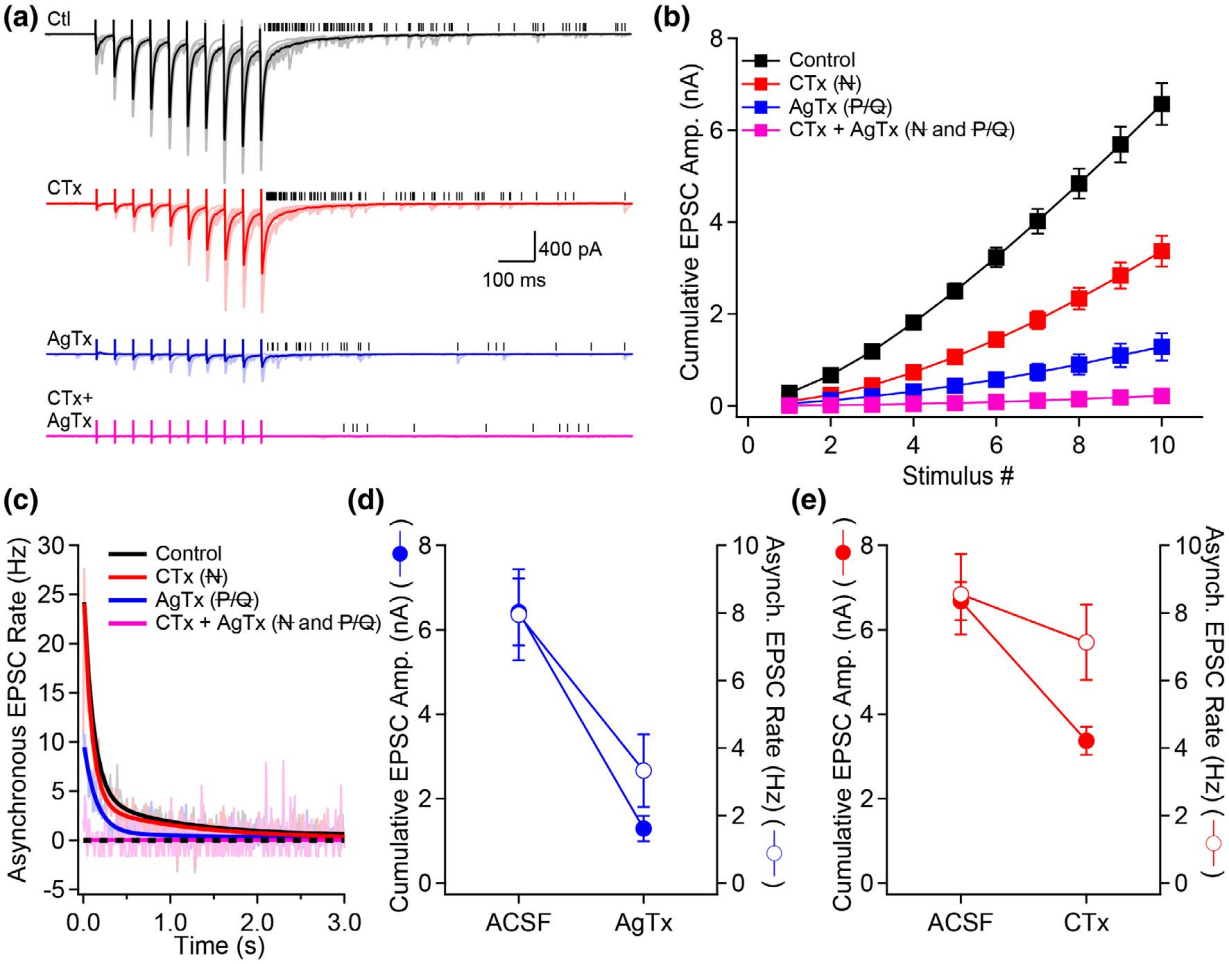
Important contribution of P/Q‐type VGCCs to asynchronous release. (a) Examples of voltage‐clamp recordings from CA3 pyramidal cells and electrical MF stimulation (10 stimuli delivered at 20 Hz). Black traces correspond to the control condition. CTx (red traces) and AgTx (blue traces) were applied to block N‐ or P/Q‐ type VGCCs, respectively. Black ticks show detected asynchronous EPSCs. (b) Cumulative synchronous EPSC amplitude as a function of stimulus number. (c) Time course of asynchronous EPSC rate as a function of time following the last stimulus in the train (bins = 10 ms). Control is in black, CTx is in red and AgTx is in blue. Light‐colored traces correspond to averaged data, and full lines show a bi‐exponential fit to the data. (d) Cumulative synchronous EPSC amplitude (filled symbols) and asynchronous EPSC rate (open symbols) in control condition and in presence of AgTx. (e) Cumulative synchronous EPSC amplitude and asynchronous EPSC rate for experiments in control and in the presence of CTx. Experiments were performed in ACSF containing 2.5 mM extracellular Ca^2+^. For all conditions tested, asynchronous release was measured in a 500 ms window starting 10 ms after the last stimulus. Number of replicates: *n* = 36 for control; *n* = 21 for CTx; *n* = 15 for AgTx

First, we performed presynaptic Ca^2+^ imaging in control condition and after the application of ω‐Agatoxin IVA (AgTx, to block P/Q‐type VGCCs) or ω‐Conotoxin GVIA (CTx, to block N‐type VGCCs) (Figure 6a). We observed that 1 AP‐evoked presynaptic Ca^2+^ transients were diminished by the application of AgTx (control: 4.3 ± 0.6% ΔG/R; AgTx: 2.4 ± 0.4% ΔG/R; *n* = 11, *p* < .001) or CTx (control: 5.2 ± 0.7% ΔG/R; CTx: 3.5 ± 0.3% ΔG/R; *n* = 9, *p* < .01) (Figure 6b‐c). Application of AgTx produced a significantly larger inhibition of presynaptic Ca^2+^ transients, an observation consistent with previous reports. Last, we investigated how P/Q‐ and N‐type VGCCs control asynchronous release. We have previously shown that P/Q‐ and N‐type VGCCs possess distinct roles in short‐term facilitation observed at the MF–CA3 synapse (Chamberland et al., 2017). However, the role of multiple Ca^2+^ sources in controlling distinct modes of release remains generally unknown. Trains of EPSCs were evoked by 10 stimuli at 20 Hz and AgTx or CTx were applied (Figure 7a). Synchronous release was significantly decreased by the application of either toxin, with AgTx having a more pronounced effect, a result which was consistent with the observation that P/Q‐type VGCCs contribute a larger fraction of AP‐evoked Ca^2+^ flux in MF boutons (Figure 7b). We next measured asynchronous release following the termination of the stimulus train. Surprisingly, these measurements revealed that while AgTx strongly decreased asynchronous release, CTx had virtually no effect on the frequency and time course of asynchronous release (Figure 7c). The effects of AgTx and CTx on synchronous and asynchronous release were next contrasted. While AgTx decreased synchronous and asynchronous release similarly (Figure 7d), CTx had a much more pronounced effect on synchronous release. Indeed, the decrease observed in asynchronous release was small and statistically nonsignificant (Figure 7e). Thus, these findings reveal that P/Q‐ and N‐type VGCCs contribute differently to synchronous and asynchronous release at MF–CA3 synapses. Moreover, while N‐type VGCCs contribute a significant fraction of presynaptic Ca^2+^ elevations, their role in mediating asynchronous release remains minor and nonsignificant.

### 2.7 Increasing intracellular Ca^2+^ buffering decreases the initial magnitude but lengthens asynchronous release

Our results implicate that slow Ca^2+^ removal from MF boutons gates long‐lasting asynchronous release. We next performed experiments to explore how manipulating the intracellular Ca^2+^ buffering dynamics would alter asynchronous release.

**FIGURE 8:**
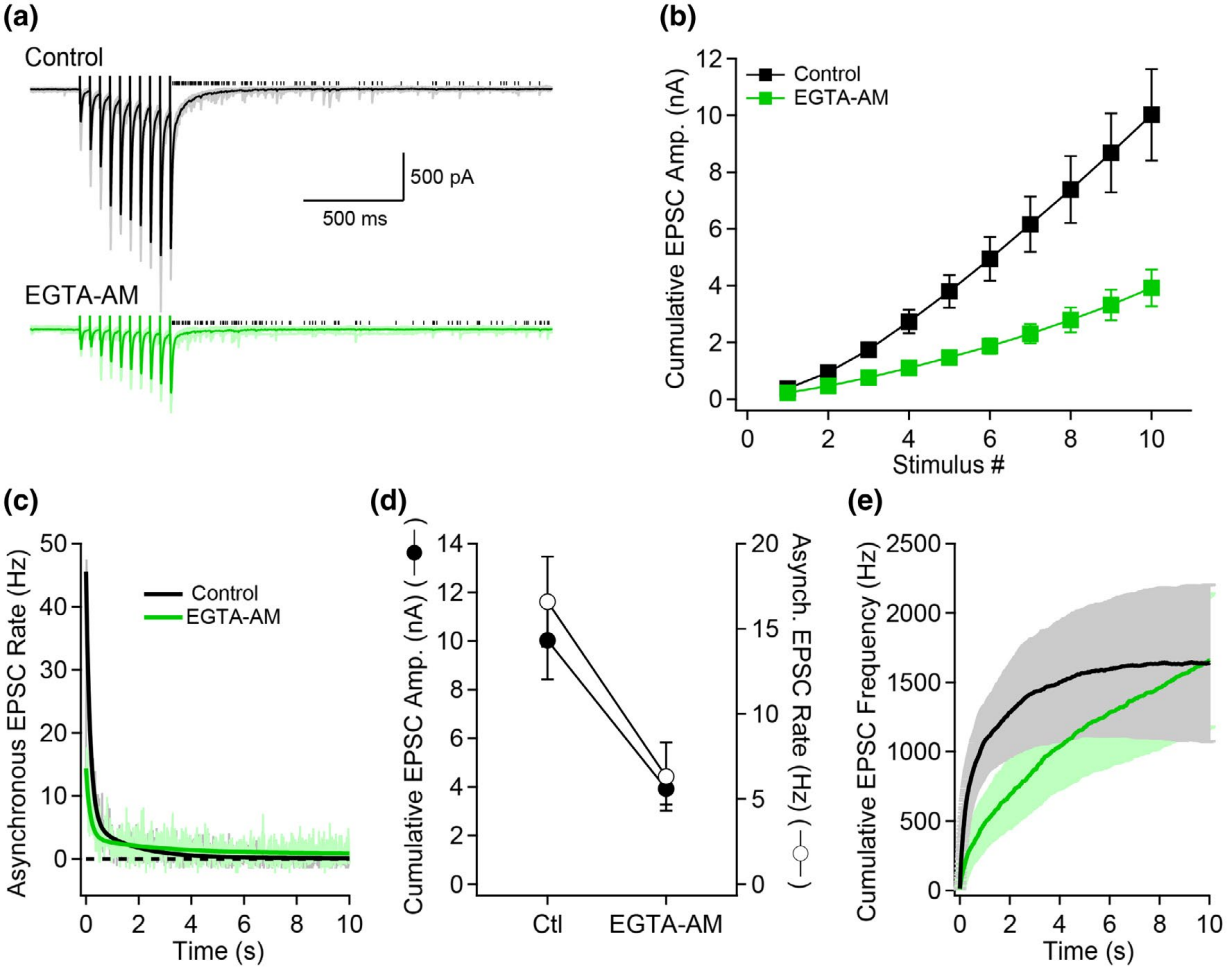
EGTA–AM prolongs the time course of asynchronous release. (a) Voltage‐clamp recordings in control condition (black) and in presence of EGTA–AM (green). (b) Cumulative synchronous EPSC amplitude as a function of stimulus number. (c) Asynchronous EPSC rate as a function of time for control and EGTA–AM conditions. (d) Cumulative synchronous and asynchronous release as a function of the recording condition. Asynchronous release was measured over a 500 ms window starting 10 ms after the last stimulus. (e) Cumulative asynchronous release as a function of time. This result demonstrates that while asynchronous release is initially lower in EGTA–AM, its duration is lengthened so that the total asynchronous release becomes similar after 10 s

Application of the slow Ca^2+^ chelator EGTA–AM is expected to reduce the initial frequency of asynchronous release, but should make asynchronous release longer as it lengthens Ca^2+^ decay from the bouton (Helmchen & Tank, 2015). First, EGTA–AM greatly decreased both synchronous and asynchronous release by similar fractions (*n* = 8, Figure 8a–d). While the initial phase of asynchronous release was drastically decreased by EGTA–AM, the asynchronous release total duration was largely extended, such that similar levels of asynchronous release were reached 10 s following the termination of the stimuli trains (*n* = 8, Figure 8e). These results suggest that asynchronous release is initially decreased but then prolonged, such that the total asynchronous release observed over 10 s is similar as in control.

## 3 DISCUSSION

Our results show that atypical slowly decaying presynaptic Ca^2+^ elevations in MF presynaptic terminals contribute to long‐lasting asynchronous release following termination of granule cell firing. High‐frequency activity highly favors asynchronous release over synchronous release, and asynchronous release is preferentially mediated by P/Q‐type VGCCs over N‐type VGCCs.

### 3.1 Long‐lasting asynchronous release at MF–CA3 synapses

Asynchronous release has been shown to co‐occur with synchronous release at multiple synapses, and there is general agreement that asynchronous release increases with the level of activity (Atluri & Regehr, 1998). Asynchronous release far outlasts synchronous release, and may last for hundreds of milliseconds (Atluri & Regehr, 1998). We chose to measure asynchronous release in a window starting after the termination of synchronous release to obtain a measure independent of possible contamination by synchronous release. Here, we show an example of particularly long‐lasting asynchronous release. Under high release probability conditions or in response to high‐frequency stimulation, asynchronous release decays slowly, lasting up to seconds following stimuli termination. What are the factors that determine the duration of asynchronous release? Previous studies have explored the main determinants of asynchronous release at synapses formed by cerebellar granule cells (Atluri & Regehr, 1998), at the Calyx of Held (Sakaba, 2006) and in cultured glutamatergic neurons (Otsu et al., 2004). These studies have highlighted the role of presynaptic Ca^2+^ dynamics, vesicle functional heterogeneity, and vesicle availability in controlling asynchronous release. Thus, it is possible that an interplay between Ca^2+^ dynamics and vesicle heterogeneity (Chamberland & Toth, 2016) could control the temporal dynamics of asynchronous release.

The computational modeling reproduced the general dependence of asynchronous release on the stimulation frequency. However, we note that experimentally induced asynchronous release was longer lasting than in the simulated data. One possible reason for this discrepancy is our usage of a release model, which was originally constrained for the calyx‐of‐Held synapses (Sun et al., 2007). It is possible that binding affinities of Ca^2+^ release sensors involved in fusion machinery in MF boutons differ from those in the calyx‐of‐Held terminals, and this is reflected in a faster decay of the asynchronous release rates in our in silico data. Furthermore, the models of Ca^2+^‐activation of vesicular release developed in the calyx of Held are based on measuring the rate of vesicular release to stepwise increases of [Ca^2+^] using Ca^2+^‐uncaging (Lou, Scheuss, & Schneggenburger, 2005; Sun et al., 2007). Thus, these models do not take into account a possible slow reverse rate of Ca^2+^ activation of asynchronous release. Indeed previous work demonstrated that Ca^2+‐^dependent enhancement of synchronous release outlasted the removal of Ca^2+^ from the MFB terminals (Regehr, Delaney, & Tank, 1994). Despite that observation, computational modeling qualitatively replicated the experimental data supporting our main conclusions.

Experiments with EGTA–AM provide insights into the mechanisms controlling the duration of asynchronous release at the MF–CA3 synapse. EGTA–AM decreased both synchronous and asynchronous release, but drastically lengthened asynchronous release so that the total asynchronous release became similar to control 10 s following the stimulation train. As more Ca^2+^ is extruded from the bouton, Ca^2+^ unbinds from EGTA and contribute to sustained asynchronous release. This finding suggests that one of the main determinants of asynchronous release in MF terminals is the duration of Ca^2+^ elevations.

### 3.2 Slow‐decaying presynaptic Ca^2+^ transients in MF terminals

The amplitude and duration of presynaptic Ca^2+^ transients are key parameters driving the release of synaptic vesicles. Our results show that presynaptic Ca^2+^ elevations in MF terminals are long‐lasting, and with a measured Ca^2+^ removal rate of 0.2–0.4 ms^−1^, a value on the order of 10 times slower than in small presynaptic terminals from dentate granule cells (Jackson & Redman, 2003). These results are consistent with previous observation at this synapse (Chamberland et al., 2018; Scott & Rusakov, 2006). The physiological basis of such slowly decaying presynaptic Ca^2+^ transients remain mostly unexplored, although sequestration by Ca^2+^ stores contribute to ~22% of the extrusion/buffering capacity in these boutons (Scott & Rusakov, 2006). The slow decay of Ca^2+^ transients could also be explained by a low surface‐to‐volume ratio of these boutons, as most of Ca^2+^ extrusion mechanisms are found at the membrane. In addition, a combination of high‐affinity mobile and immobile Ca^2+^ buffers could contribute to prolong the decaying phase of Ca^2+^ elevations (Chamberland et al., 2018; Vyleta & Jonas, 2014). An aspect of the work presented here is the presence of a bi‐exponential decay for Ca^2+^ elevations. Such bi‐exponential has previously been observed in cortical axons (Koester & Sakmann, 2000), and more recent work has shown that Ca^2+^ elevations in hippocampal CA1 pyramidal neurons boutons also demonstrate a bi‐exponential decay (Hamid, Church, & Alford, 2019). The later study showed that this bi‐exponential decay can be attributed to Ca^2+^ buffering and extrusion in boutons. Therefore, such mechanisms contributing to lengthen the duration of presynaptic Ca^2+^ elevations in MF boutons are likely to impact asynchronous release.

### 3.3 Multiple pools of vesicles in MF terminals

Vesicles can be classified in pools in presynaptic terminals, with some vesicles contributing to specific types of release (Alabi & Tsien, 2012). Our work previously identified that asynchronous release from MF terminals is preferentially mediated by a population of adaptor protein‐3 (AP‐3) expressing vesicles, which are preferentially recycled via bulk endocytosis (Evstratova et al., 2014). Recent findings further demonstrated that endocytosis operates in two modes at hippocampal mossy fiber synapse, with brief stimulation evoking fast clathrin‐independent endocytosis while stronger stimulation train evoked a slower mode of clathrin‐dependent endocytosis (Delvendahl, Vyleta, von Gersdorff, & Hallermann, 2016). Considering that asynchronous release is preferentially evoked by strong stimulation trains, vesicles endocytosed through the clathrin‐independent mechanism could be involved in asynchronous release. We note that AP‐3 deficiency did not alter short‐term facilitation of synchronous responses at MF synapses (Evstratova et al., 2014), thus, arguing that vesicles mediating synchronous and asynchronous release modes might originate from different pools. On the contrary, it has been shown that the high‐affinity Ca^2+^ sensor synaptotagmin‐7 is involved in short‐term facilitation at multiple synapses including the hippocampal MF (Jackman, Turecek, Belinsky, & Regehr, 2016), a sensor also associated with asynchronous release (Bacaj et al., 2013). Interestingly, it has been shown in cerebellar granule cells that synaptotagmin‐7 is a sensor for both short‐term facilitation and asynchronous release (Turecek & Regehr, 2018). Whether the same Ca^2+^ sensor contribute to exocytosis of vesicles originating from distinct pools to mediate short‐term facilitation and asynchronous release at MF synapses remains to be determined.

### 3.4 Distinct functions of P/Q‐ and N‐type VGCCs in control of release

Previous reports indicated that distinct Ca^2+^ sources can selectively contribute to specific modes of neurotransmitter release (Fawley, Hofmann, & Andresen, 2016; Wen et al., 2013). The results reported here show that Ca^2+^ influx through P/Q‐type VGCCs is a strong mediator of asynchronous release compared to N‐type VGCCs at the MF synapse. Our previous results supported the idea that P/Q‐type VGCCs are loosely coupled to vesicles in MF terminals, while N‐type VGCCs are in a tight‐coupling configuration (Chamberland et al., 2017). Ca^2+^ influx through N‐type VGCCs mediate a significant fraction of release that plateaus as a function of release probability, while Ca^2+^ influx through P/Q‐type VGCCs largely contribute to short‐term facilitation (Chamberland et al., 2017). Tight‐coupling is typically associated with reliable neurotransmission while loose‐coupling is generally thought to allow integration of recent synaptic activity over longer timescales. This is because tightly coupled vesicles located near VGCCs sense high‐concentration nano/microdomain Ca^2+^ elevations, which efficiently trigger synchronous release before rapidly collapsing (Ermolyuk et al., 2013). On the contrary, vesicles located more distant to the VGCCs are unlikely to fuse following a single AP‐mediated Ca^2+^ influx event, due to insufficient Ca^2+^ concentration in their vicinity. Therefore, loose‐coupling mediates vesicle release as a function of integrated Ca^2+^ activity over time. In contrast to synchronous release, asynchronous release is a function of the global Ca^2+^ increase. The larger contribution of P/Q‐type VGCCs to asynchronous release can be attributed to their larger relative contribution to Ca^2+^ transients, yet, it is not excluded that their relative position to synaptic vesicles also contribute to this role. For example, whether loosely coupled P/Q‐type VGCCs have privileged access to distinct pool(s) of vesicles remains unknown.

Another factor could complicate our interpretation. While we find that Ca^2+^ transients are sustained during trains, we cannot exclude that a combination of factors acting in opposite direction could maintain a sustained Ca^2+^ transients’ amplitude. For example, we cannot exclude the possibility that the Ca^2+^ flux through P/Q‐ and N‐type VGCCs may be modulated during trains by activity‐ or Ca^2+^‐dependent modulation of channels. If present, such modulation of channels would complicate our interpretations for the proposed roles of P/Q‐ and N‐type VGCCs in asynchronous release.

Could other types of VGCCs contribute to asynchronous release? Previous work demonstrated that R‐type VGCCs contribute to Ca^2+^ influx at this synapse (Li et al., 2007) and that R‐type VGCCs are involved in release during long‐lasting and high‐frequency stimulation (Dietrich et al., 2003). Here, we show that over the activity regime tested, combined application of AgTx and CTx was sufficient to block both synchronous and asynchronous release.

### 3.5 Multiplexed information transfer

Information transfer at the hippocampal MF–CA3 synapse relies on a counting code (Chamberland et al., 2018), which is independent of the granule cell firing frequency. This could serve as a mechanism for burst detection, making the presence of a burst mandatory under normal release probability to enforce information propagation. It has been observed that granule cells fire APs in burst with highly varying frequency in vivo (Pernia‐Andrade & Jonas, 2014). Our observation that asynchronous release is strongly dependent on burst frequency may suggest that the frequency component is not lost from the granule cell to CA3 pyramidal cell dialog, as it is transmitted through asynchronous release.

How does long‐lasting asynchronous release contribute to information transfer between the dentate gyrus and the CA3 region? It has previously been shown that asynchronous release from granule cells controls the precise timing of the action potential in CA3 pyramidal neurons (Evstratova et al., 2014). Thus, action potential counting could enforce the transfer of information, while high‐frequency burst could refine action potential timing. Another possible scenario is that asynchronous release maintains the postsynaptic neurons in an elevated state of activity, such that the passage of a high‐frequency burst will influence the network for several seconds. This scenario would be consistent with a recently discovered phenomenon at the granule cell to CA3 interneurons synapses. At MF to CA3 interneuron synapses, the passage of an AP burst in granule cells induces a novel form of long‐lasting facilitation in the CA3 inhibitory network (Neubrandt et al., 2018). More precisely, a short burst of granule cell firing specifically maintains MF to interneuron synapses in a state of elevated release probability over several seconds, which will cause an enhanced recruitment of interneuron in response to subsequent granule cell firing (Neubrandt et al., 2018). Thus, it is interesting to speculate that the passage of a burst maintains a select subset of target CA3 pyramidal neurons in an elevated state of excitability in preparation of subsequent activity, but simultaneously prepares the inhibitory network to provide powerful lateral inhibition (Neubrandt, Olah, Brunner, & Szabadics, 2017).

## 4 MATERIALS AND METHODS

### 4.1 Acute hippocampal slices preparation

All experiments involving the use of animals were performed in accordance with Université Laval's guidelines for animal welfare. C57BL/6 mice (P17–P30) of either sex were anesthetized with isoflurane. Following decapitation, the brain was rapidly extracted and immersed in ice‐cold cutting solution saturated with 95% O_2_ and 5% CO_2_ and containing (in mM): NaCl 87, NaHCO_3_ 25, KCl 2.5, NaH_2_PO4 1.25, MgCl_2_ 7, CaCl_2_ 0.5, glucose 25, and sucrose 75 (pH = 7.4, 330 mOsm). The brain was hemisected and dissected on a petri dish while immersed in the cutting solution and was oriented to preserve the MF axons (Bischofberger, Engel, Li, Geiger, & Jonas, 2006). Both hemispheres were glued on the platina of a VT1000S Vibratome (Leica, Germany) and 300 µm slices were prepared. Slices were transferred to a heated (32°C) and oxygenated solution of artificial cerebrospinal fluid (ACSF) containing (in mM): NaCl 124, NaHCO_3_ 25, KCl 2.5, MgCl_2_ 1.2, CaCl_2_ 2.5, and glucose 10 (pH = 7.4, 300 mOsm) for 30 min, at which time the incubator was turned off and the slices were allowed to recover for an additional 30 min.

### 4.2 Electrophysiology

Acute slices were transferred to a recording chamber mounted on an upright microscope. Slices were held under a U‐shaped harp and perfused with oxygenated ACSF (described above) at a speed of 2–3 ml/min. For experiments performed in 1.2 mM Ca^2+^, the Mg^2+^ concentration was adjusted to 2.5 mM. The Mg^2+^ concentration was reduced to 0 mM for experiments performed in 6 mM Ca^2+^. Whole‐cell recordings were performed at 32°C. CA3 pyramidal cells were visually identified using a 40× (0.8 NA, Olympus) water‐immersion objective. Microelectrodes were pulled from borosilicate filaments and filled with an intracellular solution containing (in mM): K‐gluconate 120, KCl 20, HEPES 10, MgCl_2_ 2, Mg_2_ATP 2, NaGTP 0.3, phosphocreatine 7, EGTA 0.6 (pH = 7.2, 295 mOsm). CA3 pyramidal cells were voltage‐clamped at −70 mV. The MF were stimulated by positioning an electrode connected to a stimulus isolator (A360, WPI, Florida, USA) in *stratum lucidum*. The stimulating electrode was moved along *stratum lucidum* until facilitating EPSCs with rapid kinetics were identified.

### 4.3 Drugs

Selective toxins for Ca_V_2.1 and Ca_V_2.2 were used to block and investigate the role of Ca^2+^ flux through P/Q‐ and N‐type VGCCs in asynchronous release. P/Q‐type VGCCs were blocked with 200 nM ω‐Agatoxin IVA (AgTx, Alomone Labs, Israel) and N‐type VGCCs were blocked with 200 nM ω‐Conotoxin GVIA (CTx, Alomone Labs, Israel). In all electrophysiology and Ca^2+^ imaging experiments, the brain slices were perfused for 10 min with the toxins to allow full diffusion and complete blockade of VGCCs. Following recordings, MF identity was confirmed by application of DCG‐IV (1 µM, Tocris/R&D Systems, MN, USA).

### 4.4 Two‐photon presynaptic calcium imaging

MF bouton Ca^2+^ imaging was performed on a custom‐built random‐access (RAMP) two‐photon microscope operated in LabView (Karthala Systems, France) (Chamberland et al., 2014; Otsu et al., 2008). This imaging equipment allowed to obtain a full spatial profile of Ca^2+^ elevations in MF boutons, while maintaining a high temporal resolution. The laser beam from a Ti‐Sapphire laser (Chameleon Ultra II, 80 MHz repetition rate, pulse width 140 fs, tuned at 800 nm, Coherent, CA, USA) was directed to the back aperture of the water‐immersion objective (0.95 NA, Leica) by a pair of acousto‐optic deflectors (AODs, A–A Opto‐Electronics, France). Emitted photons were collected through an oil condenser (NA = 1.4) and short‐pass filtered at 720 nm (Semrock, NY, USA). Photons were separated in two channels based on their wavelength by a 580 nm dichroic mirror (Semrock, NY, USA) and band‐pass filtered at 500–560 nm in the green channel (Semrock, NY, USA) or 595–665 nm for the red channel. Photons were detected by external GaAsP photomultiplier tubes in both channels (H7422P‐40, Hamamatsu, SZK, Japan). The data were recorded with a custom‐written software in LabView and recorded on a personal computer. For Ca^2+^ imaging experiments, the intracellular solution was supplemented with the morphological indicator Alexa‐594 (40 µM) and the calcium indicator Fluo‐5F (385 µM) or Fluo‐4FF (375 µM). Whole‐cell recordings were obtained from visually identified granule cells using a 25X Leica objective (NA = 0.95). The morphological indicator and the calcium‐sensitive dye were left to passively diffuse in the granule cell axon for 1 hr before starting recordings. Granule cells with cut axons axon were discarded. For neurons with an intact axon, it was followed to the CA3 region where giant MF boutons could be identified (Figure 4a). The bouton was zoomed in and points of interest were selected to homogenously cover the bouton, and thus, obtain a full recording of Ca^2+^ dynamics in the whole bouton (Figure 4b1,2). Single of bursts (10 at 20 Hz) of action potentials were evoked by brief current injection at the soma. The resulting presynaptic Ca^2+^ transients were recorded and averaged over 20 trials for experiments performed with Fluo‐5F and for 40 to 90 trials for experiments performed with the low‐affinity indicator Fluo‐4FF. Individual trials were interspaced by 30 s to avoid photo‐damage and photo‐toxicity.

### 4.5 Nonstationary single compartment model of presynaptic Ca^2+^ dynamics

The model is described by the following system of differential equations:

$$\begin{array}{c} \frac{d[Ca^{2+}]}{dt}=j_{Ca}+k_{off}^{I}[CaI]‐k_{on}^{I}[Ca^{2+}][I]+\sum_{i}^{}(k_{off}^{B_{i}}[CaB_{i}]‐k_{on}^{B_{i}}[Ca^{2+}][B_{i}])‐P_{rem}\\ \frac{d[I]}{dt}=k_{off}^{I}[CaI]‐k_{on}^{I}[Ca^{2+}][I]\\ \frac{d[B_{i}]}{dt}=k_{off}^{B_{i}}[CaB_{i}]‐k_{on}^{B_{i}}[Ca^{2+}][B_{i}] \end{array}$$

where the square brackets denote concentrations, and the superscript indices of the reaction rate constants denote endogenous Ca^2+^ buffers *B_i_* or the Fluo‐4FF indicator *I*. The AP‐dependent Ca^2+^ influx time course *j_ca_* was approximated by the Gaussian function $j_{Ca}=\frac{\Delta [Ca^{2+}]total}{\sigma \sqrt{2\pi }}\sum_{i}^{}\exp\left(‐\frac{(t‐t_{i}^{AP})2}{2\sigma^{2}}\right)$, where $t_{i}^{\mathrm{AP}}$ denotes the times of peaks of Ca^2+^ currents during each action potential. To take into account our experimental conditions ${\left[Ca^{2+}\right]}_{\mathrm{ext}}=$ 2.5 mM (in comparison to ${\left[Ca^{2+}\right]}_{\mathrm{ext}}=$ 1.2 mM used in (Chamberland et al., 2018)), we increased $\Delta {\left[Ca^{2+}\right]}_{\mathrm{total}}$ by a factor 1.85 (estimated from (Weber et al., 2010)) from $\Delta {\left[Ca^{2+}\right]}_{\mathrm{total}}$=33.33 µM to $\Delta {\left[Ca^{2+}\right]}_{\mathrm{total}}$=61.7 µM. As in (Chamberland et al., 2018) Ca^2+^ removal was approximated by a first‐order reaction $P_{\mathrm{rem}}=k_{\mathrm{rem}}\left(\left[Ca^{2+}\right]‐{\left[Ca^{2+}\right]}_{\mathrm{rest}}\right)$. We assumed that a MF terminal contains three endogenous buffers (calbindin‐D_28K_, calmodulin, and ATP). The complete set of model parameters and Ca^2+^ binding reactions, including concentrations and binding properties of the endogenous buffers can be found in (Chamberland et al., 2018). The model was numerically solved using the adaptive step‐size Runge–Kutta algorithm.

### 4.6 Modeling of Ca^2+^‐triggered synaptic vesicle fusion

To simulate glutamate release, we used *[Ca* *^2+^* *](t)* profiles from the single‐compartment Ca^2+^ dynamics model in the stochastic simulations (implemented in MATLAB using the modified Gillespie's algorithm (Anderson, 2007; Gillepsie, 1977)) based on the dual‐Ca^2+^‐sensor model (Sun et al., 2007) (Figure 5a). The release model also contained a stochastic re‐priming step, which was preceded by a short refractory period (1 ms) immediately after vesicle fusion. The model parameters were as in (Sun et al., 2007); α = 153 (μM s)^−1^, β = 5,800 s^−1^, b = 0.25, χ = 2.94 (μM s)^−1^, δ = 130 s^−1^, $\gamma_{1}$ = 0.417 × 10^‐3^ s^−1^, $\gamma_{2}$ = 6,000 s^−1^, $\gamma_{3}$ = 6,000 s^−1^. The repriming rate was constrained using the experimental release rate values at the start of asynchronous release (10 ms after the last AP, see Figure 5b), and was used in the results simulations as *k_rep_* = 75 s^−1^. We performed 20,000 independent stochastic runs for each stimulation paradigm and the obtained data were analyzed in bins of size 10 ms.

### 4.7 Analysis of calcium imaging data

Calcium imaging data were extracted using custom‐written software in LabView and exported to Igor Pro 6.3 (Wavemetrics, Oregon, USA). Peak amplitude measurements and analysis of decay kinetics were performed in Igor Pro 6.3. Individual trials were first averaged in space for all points recorded in the bouton and averaged across trials. Amplitude of Ca^2+^ transients were measured from the averages. AgTx and CTx were allowed to diffuse for 10 min in the recording chamber before recordings were performed. Examples shown in figures are unprocessed and not filtered.

### 4.8 Analysis of electrophysiological data and statistical analysis

Spontaneous and asynchronous EPSCs were automatically detected using the Clampfit software Event Detection module. Asynchronous release was measured in a 500 ms window starting 10 ms after the last stimulus termination. The average frequency of spontaneous EPSCs, measured before the stimulation train, was subtracted from the frequency of spontaneous EPSCs following the train to obtain the asynchronous component of release. Peak synchronous EPSC amplitudes were measured from averaged trials in Clampfit. Statistical analysis was performed with paired and unpaired Student's *t* test. Differences were deemed significant if *p* < .05.

## AUTHOR CONTRIBUTIONS

S. Chamberland, Y. Timofeeva, K. Volynski, and K. Tóth designed the study. S. Chamberland, A. Evstratova, and K. Tóth performed experiments, Y. Timofeeva, C. A. Norman, and K. Volynski performed computational modeling, S. Chamberland, Y. Timofeeva, K. Volynski, and K. Tóth analyzed the data, S. Chamberland, Y. Timofeeva, K. Volynski, and K. Tóth wrote the paper.
